# The workplace violence against emergency nurses in Jordan: its impact on the treatment process and nursing work

**DOI:** 10.1186/s12912-026-05224-4

**Published:** 2026-08-08

**Authors:** Mohammad Ghassan Ibrahim, Hana Mohammad Abu-Snieneh

**Affiliations:** 1 Faculty of Nursing, Al-Zaytoonah University of Jordan, Amman, Jordan; 2 Examinations Department, Jordanian Nursing Council (JNC), Amman, Jordan

**Keywords:** Emergency nurses, Jordan, Non-physical violence, Physical violence, Workplace violence

## Abstract

**Background:**

The Jordanian emergency nurses are now the victims of cruel behaviors such as violence. The incident of Workplace Violence (WPV) is increasing annually with a growing impact on the continuity of the treatment process and nursing work.

**Aim:**

The purpose of this study was to identify the types of WPV against nurses in emergency departments EDs and identify the contributing factors. Also, it establishes the variations in WPV exposure among ED nurses based on the geographical location of hospitals (North, Middle, and South).

**Methods:**

A descriptive cross-sectional, comparative design was conducted with a convenience sample of 200 emergency nurses. Data were collected via the Arabic version of the WHO workplace violence questionnaire. Bivariate associations were examined using Chi-square, Mann-Whitney U, and Kruskal-Wallis tests, with Cramer’s V for effect sizes. Binary Logistic Regression was executed to identify independent predictors of violence exposure (*P* ≤ 0.05).

**Results:**

Overall, 73.5% of emergency nurses experienced workplace violence; 70% faced non-physical violence and 27% experienced physical violence. Bivariate analysis showed male nurses were significantly more exposed to physical violence than females (*p* = 0.024). Binary logistic regression revealed that female gender significantly predicted lower odds of physical violence (OR = 0.42,95% C.I.[0.21, 0.83], *p* = 0.01). Significant geographical variations were found for both physical (x² = 6.112, *p* = 0.047) and non-physical violence(x² = 15.755, *p* = 0.001), with the Middle region exhibiting the highest exposure rates. Working in the Southern region significantly predicted lower odds of non-physical violence (OR = 0.56, 95% C.I.[0.37, 0.87], *p* = 0.01).

**Conclusion:**

Workplace violence is highly prevalent among Jordanian emergency nurses, significantly disrupting the continuity of the treatment process and compromising nursing work environments. Aligned with the study’s aim, the findings highlight that hospital overcrowding and regional density—particularly in the Middle region—escalate violence exposure. Urgent administrative interventions, such as extending outpatient clinic hours and strengthening security enforcement, are vital to safeguard emergency staff and optimize healthcare delivery.

**Supplementary Information:**

The online version contains supplementary material available at 10.1186/s12912-026-05224-4.

## Introduction

Violence is the third leading origin of occupational death and the second main reason of increased female mortality in the United States. Violence is a problem in various professions, including healthcare; the greatest rates of violence were seen in the emergency department (ED) and among health workers. Nurses face a significant threat of violence in several countries around the world [1]. Violence in the ED affects roughly 92.9% of Taiwanese nurses and 73.2% of Malaysian nurses [1]. Additionally, 66% of Canadian emergency healthcare providers reported being exposed to violence at work. Furthermore, Arab countries report workplace violence. In Egypt, the majority of nurses in ED (85.6%) had experienced Workplace violence (WPV) [2].

As per the World Health Organization (WHO), WPV has become an alarming global phenomenon, with health sector violence accounting for nearly a quarter of all workplace violence incidents worldwide [3]. This significantly highlights that healthcare providers face a disproportionately higher risk of violence compared to workers in other occupational settings [3].

WPV has an influence not only on healthcare quality but also on nurse work satisfaction [4].

Darawad et al., (2015) [5] conducted a study in Jordan to quantify the incidence of violence in EDs, and the results showed most emergency nurses (91.4%) of the 174 study among 300 participants faced one or more types of WPV, with workload and patient overcrowding being the most common causes. Later, Higazee et al., (2017) [6] undertook another study in Jordan’s capital city, Amman. The findings pointed out that almost half of the 107 participants complained of WPV. The authors advocated boosting staff numbers to prevent his phenomenon in Jordanian hospitals.

Increased turnover rates among emergency nurses have been noted as one of the major consequences of WPV in recent years [7]. Likewise, the broader healthcare system suffers from WPV-related complications, including low patient satisfaction, an inability to offer evidence-based treatment, insecure work environments, and severe financial struggles [7]. For instance, ED violence costs Ontario alone $23.8 million each year [7].

The aim of this research is to specify the types and determine the associated factors of workplace violence among emergency nurses in Jordan. In addition, it determines the differences in exposure to WPV among ED nurses based on the geographical location of hospitals (North, Middle, and South).

The primary objective of this study is to assess the prevalence and potential associated factors of physical and non-physical workplace violence (WPV) among emergency nurses in Jordanian hospitals. Specifically, this study tests the following research hypotheses:

### Hypothesis 1 (H_1)

There are statistically significant differences in exposure to physical and non-physical WPV based on nurses’ demographic and professional characteristics.

### Hypothesis 2 (H_2)

Geographical distribution (North, Middle, and South regions) significantly influences the patterns and rates of exposure to physical and non-physical WPV among emergency nurses.

## Methodology

### Research design

This study employed a descriptive, cross-sectional, comparative research design. This design was selected to simultaneously evaluate the prevalence of workplace violence and statistically compare the exposure rates and contributing factors across different geographical regions (North, Middle, and South) in Jordan.

This study was conducted and reported in adherence to the Strengthening the Reporting of Observational Studies in Epidemiology (STROBE) guidelines for cross-sectional studies, ensuring transparent and comprehensive reporting of the methodology and findings.

### Population

The target population refers to all Jordanian emergency nurses working in EDs. On the other hand, the accessible population is the emergency nurses with one year of experience in EDs in the six selected hospitals at Ministry of Health (MOH).

### Sample and sampling

To ensure geographic representation across Jordan, a convenience sampling approach was utilized to recruit 200 emergency nurses from emergency departments distributed across the three main geographical regions: North, Middle, and South. This regional distribution allowed for a comparative analysis between different geographical locations while maintaining the feasibility of data collection through a convenience sample of nurses who met the inclusion criteria [8]. Therefore, all suitable nurses were asked to participate.

#### Sample size

The sample size was determined prior to data collection to ensure adequate statistical power. Using G*Power software (version 3.1.9.7), a power analysis for logistic regression was conducted. Based on a significance level **(α)** of 0.05 (two-tailed), a medium effect size, and a statistical power (1-β) of 0.80, the analysis indicated a minimum requirement of 98 participants. To account for potential attrition and to enhance the robustness of the study findings, the target sample size was increased. Ultimately, the study successfully recruited 200 emergency nurses, a sample size that exceeds the calculated minimum, thereby providing sufficient statistical power to detect meaningful associations and ensuring the stability of the binary logistic regression models.

### The inclusion and exclusion criteria

#### Inclusion criteria

The study included all registered nurses currently working full-time in the emergency departments (EDs) of the selected government hospitals with a minimum of one year of experience in the ED.

#### Exclusion criteria

Emergency nurses with less than one year of experience in the ED were excluded from the study. Additionally, practical nurses, nurse interns, nursing students, and those on extended administrative or sick leave during the data.

### Setting

This study was conducted from June 2022 to July 2022. Data were collected within the EDs of six governmental hospitals distributed across the North, Middle, and South regions of Jordan, ensuring a representative sample that captures the influence of WPV on the treatment process and nursing work across various healthcare settings.

### Study Instrument

#### Questionnaire

In this study, data were collected using the Arabic version of the “Workplace Violence in the Health Sector Country Case Study Questionnaire”. This instrument was originally developed by the Joint Programme of the World Health Organization (WHO), the International Labour Office (ILO), the International Council of Nurses (ICN), and Public Services International (PSI). The validated Arabic version used in this study was adopted from Alshehri (2017) [9], which was based on the work of Kitaneh and Hamdan (2012) [10]. The questionnaire is structured into four main sections: (1) Socio-demographic and workplace characteristics, (2) Physical workplace violence, (3) Non physical workplace violence (including verbal abuse, bullying/mobbing, and sexual harassment), and (4) Responses and actions taken following violence incidents.

**The concluding version of the questionnaire contained four sections**:

##### Section one

the demographic data of the participants, including age, gender, qualification, occupation, experience as a nurse and as an emergency nurse.

##### Section two

“physical violence”, eight questions aimed to uncover the experiences of physical violence (multiple choice).

##### Section three

“non-physical violence”, eight questions aimed to uncover the experiences of non-physical violence (multiple choice).

##### Section four

means of protection, systems and policies available about the violence against workers in the hospitals, ten questions. (multiple choice)

At the end two questions were added to identify society culture’s role in increasing violence. The first question is: “Does community’s culture contribute in the workplace violence?” “Yes,” “no,” or “I do not know.” The second question is: “In the Jordanian society, what are the procedures to cope with the incident after it has occurred that “relatives of the aggressor interfered and the personal right had waived”, “the police had notified to take action”, “inter-tribal settlement accord”, or “others”?

### Validity

The instrument used in this study, the Arabic version of the ‘Workplace Violence in the Health Sector Country Case Study Questionnaire,’ has established psychometric properties. Its validity and reliability were previously confirmed by Alshehri (2017) and Kitaneh and Hamdan (2012). In the current study, the instrument’s validity was further supported by consultation with clinical and academic specialists in emergency nursing, who reviewed the items to ensure cultural relevance and clarity within the Jordanian healthcare context. Furthermore, a pilot study (*n* = 20) was conducted to confirm the clarity and cultural appropriateness of the items prior to full-scale data collection.

### Ethical considerations

Ethical approval for this study was authorized by the Al-Zaytoonah University Institutional Review Board (IRB), the Ministry of Health, the directors of each hospital, and the emergency department (ED) heads. The study was conducted in accordance with the Declaration of Helsinki. All participants provided informed consent, guaranteeing that their involvement was strictly voluntary, with no negative direct or indirect effects, and acknowledging their right to withdraw at any time without explanation. To ensure strict confidentiality and data privacy, all responses were anonymized, and no personally identifiable information was collected or stored [11].

### Piloting test

A pilot study was conducted to test the feasibility of the questionnaire and any concerns that may have developed during participant administration, coding, and scoring. Then, the researcher removed the twenty pilot study participants from the overall sample size. The questionnaire took about 10 min to complete.

### Data collection

The data collection process began in June 2022 and went through July 2022. After obtaining permission from the MOH to collect data for six government hospitals, a personal interview of each head nurse for the ED in each selected hospital was conducted to facilitate data collection through the official online group on WhatsApp or through email because the questionnaire used in this study is an electronic online questionnaire via a google form.

### Data analysis

Data were coded and analyzed using IBM SPSS Statistics version 26 (IBM, Chicago, IL, USA). A two-tailed significance level was set at *p* ≤ 0.05 with a 95% confidence interval (C.I.). The statistical analysis plan was explicitly structured to answer the study’s specific research questions as follows:

#### Statistical analysis per research objective

- **To answer Research Question 2** (Associated Factors): Bivariate inferential analyses were performed. The Chi-square (χ²) test of independence was used to compare the incidence of categorical factors across demographic profiles (note: bracketed numbers following the χ² symbol denote degrees of freedom [df] throughout the text). Non-parametric Mann-Whitney U and Kruskal-Wallis H tests were employed to compare the mean ranks of exposure when data deviated from normality. Furthermore, Cramer’s V test was used to establish the practical magnitude (effect size) of these categorical associations.
- **To answer Research Question 3 (Geographical Differences)**: Chi-square χ² and Kruskal-Wallis tests were applied to detect statistically significant differences in physical and non-physical violence exposure rates among the North, Middle, and South regions.

#### Variable coding in binary logistic regression

- A binary logistic regression analysis was conducted to identify the independent predictors of physical and non-physical workplace violence. The models included demographic and professional variables (such as gender and clinical experience) as well as geographical regions. In this modeling approach, ‘Male’ was set as the baseline reference category for gender, and the ‘Middle region’ was established as the reference category for geographical distribution. Adjusted odds ratios (OR) are reported alongside their 95% confidence intervals (Cls) and exact p-values to demonstrate the relative risk of exposure compared to these baseline categories.
- **Dependent Variables**: Were dummy-coded as dichotomous outcomes, where **1 = Yes** (Exposed to violence within the last 12 months) and **0 = No** (Not exposed).
- **Independent Predictors**: Categorical predictors were dummy-coded with designated baseline reference groups. For the gender model, **Male** was set as the reference category to evaluate the relative risk of female nurses. For the regional model, the high-density **Middle region** served as the reference baseline to contrast the relative risk reductions in the North and South regions.

#### Interpretation and statistical justifications

- **Justification of Tests**: Chi-square and non-parametric rank tests are mathematically justified due to the nominal and ordinal distribution of the survey data. Cramer’s V was utilized because p-values are highly sensitive to sample sizes; thus, Cramer’s V provides an un-biased measure of effect size strength (where ≤ 0.1 is small, 0.3 **~** is moderate). Binary logistic regression is the gold standard for predicting dichotomous outcomes while controlling for confounding variables.
- **Drawing Conclusions**: Results were interpreted using Odds Ratios (OR), unstandardized regression weights (B), and Wald statistics. An OR > 1 indicated an increased likelihood of exposure, whereas an OR < 1 demonstrated a protective effect or decreased odds of experiencing WPV compared to the baseline reference group.

## Result

Out of 328 eligible emergency nurses across the selected hospitals, a total of 200 completed and returned valid questionnaires, yielding a response rate of 60.9%. No missing data or incomplete responses were detected among the final sample (*N* = 200) due to the mandatory field settings applied in the electronic online survey.

### The sociodemographic characteristics of the participants

A total of 200 emergency nurses took part in the study. More than half of the participants fell within the 20–30 age range (*n* = 106, 58%), and males represented 55.5% (*n* = 111) of the total sample. Regarding occupational characteristics, the vast majority were registered nurses (*n* = 190, 95%), followed by head nurses (3%) and manager nurses (2%). In terms of educational qualifications, the majority held a Bachelor’s degree (*n* = 169, 84.5%), while 12.5% possessed a Master’s degree and 3% held a Diploma. Furthermore, 39.5% of the respondents had 1–5 years of total nursing experience, closely followed by those with 6–10 years of experience (35.5%). For specific emergency department (ED) tenure, nearly three-quarters of the sample (*n* = 143, 73.5%) had between 1 and 5 years of specialized ED experience (Table 1).

**Table 1:** The sociodemographic characteristics of participants (*N* = 200)

Variable	Category	*N*	%
Gender	Male	111	55.5
	Female	89	45.5
Age	20–30	106	58
	31–40	67	33
	41–50	15	7
	51 +	2	1.5
Occupation	Register nurse	190	95
	Head nurse	6	3
	Manager nurse	4	2
Qualification	Diploma	6	3
	Bachelor	169	84.5
	Master	25	12.5
Experience working as a nurse (years)	1–5	78	39.5
	6–10	71	35.5
	11–15	30	15
	16–20	21	10.5
Experience working as an emergency nurse (years)	1–5	143	73.5
	6–10	35	17.5
	11–15	13	6.5
	16–20	5	2.5

### Research questions

#### Research question 1: What are the types of WPV exposed by emergency nurses in Jordan?

The WPV, physical or non-physical or both in the last 12 months, were recorded by 147 out of 200 nurses (73.5%), 140 out of 200 (70%) reported non-physical violence and 54 out of 200 (27%) reported physical violence by emergency nurses (Table 2).

#### Research question 2: What are the associated factors for WPV against emergency nurses in Jordan?

The results showed that gender was significantly associated with the experience of physical violence (*P* = 0.024), with male participants having a higher mean rank (106.83) compared to female participants (92.60), supported by a Mann-Whitney test value of U = 4236.5.

Furthermore, there was a statistically significant difference between the geographical region and exposure to physical violence (*P* = 0.047), with a Kruskal-Wallis test value of H = 6.08 and a mean rank of 106.83 in the middle, 102.91 for the north, and 88.75 for the south region.

Regarding non-physical violence, a significant relationship was also found with the geographical region (*P* = 0.001), showing a Kruskal-Wallis test value of H = 15.67 and a mean rank of 111.61 in the middle, 103.05 for the north, and 81.35 for the south region **(**Table 2**)**.

**Table 2:** The associated factors for WPV against emergency nurses

Variable	Statistical Test & *P*-value	Physical violence				Non-physical violence			
		Yes *N* (%)	No *N* (%)	χ2	*P* .value	Yes *N* (%)	No *N* (%)	χ2	*P* .value
Gender				5.076	0.024*			0.047	0.47
Male	Mann Whitney U = 4236.5. *P* = 0.024	37 (33.3%)	74 (66.7%)			77 (69.4%)	34 (30.6%)		
Female		17 (19.1%)	72 (80.9%)			63 (70.8%)	26 (29.2%)		
Total		54 (27%)	146 (73%)			140 (70%)	60 (30%)		
Age				3.55	0.31			2.04	0.564
20–30		27 (23.3%)	89 (76.7%)			78 (67.2%)	38 (32.8%)		
31–40		23 (34.3%)	44 (65.7%)			50 (74.6%)	17 (25.4%)		
41–50		3 (20%)	12 (80%)			10 (66.7%)	5 (33.3%)		
50+		1 (50%)	1 (50%)			2 (100%)	0 (0%)		
Total		54 (27%)	146 (73%)			140 (70%)	60 (30%)		
Occupation				1.401	0.496			0.079	0.961
Register nurse		51 (26.8%)	139 (73.2%)			133 (70%)	57 (30%)		
Head nurse		1 (16.7%)	5 (83.3%)			4 (66.7%)	2 (33.3%)		
Manager nurse		2 (50%)	2 (50%)			3 (75%)	1 (25%)		
Total		54 (27%)	146 (73%)			140 (70%)	60 (30%)		
Region				6.112	0.047*			15.755	0.001**
North	Kruskal-Wallis: (Physical)H = 6.08, *P* = 0.048. (Non-Physical) H = 15.67, *P* < 0.001	15 (29.4%)	36 (70.6%)			37 (72.5%)	14 (27.5%)		
Middle		30 (33.3%)	60 (66.7%)			73 (81.1%)	17 (18.9%)		
South		9 (15.3%)	50 (84.7%)			30 (50.8%)	29 (49.2%)		
Total		54 (27%)	146 (73%)			140 (70%)	60 (30%)		

N: number %: percent P: significant level

* Significant at the 0.05 level (2-tailed)

** Significant at the 0.01 level (2-tailed)

#### The relationship between sociodemographic characteristics and WPV

The degree of association between the categorical parameters was examined using Cramer’s V test to determine the effect size of the relationships. The results showed a statistically significant, weak association between physical violence and gender (Cramer’s V = 0.159, *p* = 0.024), indicating that male nurses are more likely than female nurses to face physical violence. Furthermore, there was a statistically significant, weak association between physical violence and geographical region (Cramer’s V = 0.175, *p* = 0.047). Regarding non-physical violence, a statistically significant, moderate association was found with the geographical region (Cramer’s V = 0.281, *p* < 0.001), demonstrating that regional distribution has a stronger practical relevance and effect size on non-physical violence exposure compared to physical violence (Table 3).

**Table 3:** The relationship between participants’ sociodemographic characteristics and WPV

Sociodemographic Characteristics	Physical violence		Non-physical violence	
	Cramer’s V	*P* value	Cramer’s V	*P* value
Gender	0.159	0.024*	0.015	0.828
Age	0.133	0.31	0.101	0.564
Occupation	0.084	0.49	0.020	0.961
Qualification	0.027	0.92	0.020	0.960
Experience as a nurse	0.182	0.08	0.082	0.721
Experience in ED	0.85	0.693	0.037	0.96
Region	0.175	0.047*	0.281	0.001**

P: significant level. r: correlation

*. Correlation is significant at the 0.05 level (2-tailed)

**. Correlation is significant at the 0.01 level (2-tailed)

#### Potential associated factors for WPV

With an R2 of gender and geographical area parameters accounted for 47% of the variation in physical violence, the total binary logistic regressions modeling in linking the nurses’ experiences of physical violence was statistically significant, The Nagelkerke R² value is reported to estimate the proportion of variance in the outcome (violence exposure) explained by the predictors included in the model. Additionally, the Hosmer–Lemeshow test was used to assess model fit; a non-significant p-value (*p* > 0.05) generally indicates a good model fit, although in instances where p-values are slightly below 0.05, the model is still considered to provide a robust predictive framework for identifying risk factors.

Regarding the physical violence model, the predictors accounted for 47% of the variance (Nagelkerke R² = 0.47), and the overall model was statistically significant (χ²(1) = 38.99, *p* < 0.001). The Hosmer–Lemeshow test confirmed the model fit (χ²(4) = 3.25, *p* = 0.515).R2 geographical area factors accounted for 34% of the variation in non-physical violence, and the whole modeling was statistically significant in linking the nurses’ experiences of non-physical violence, (χ²(1) = 30.15,*p* < 0.001). Proportional odds estimates were used after the Hosmer Lemeshow test revealed that the model was fit, (χ²(1) = 8.27, *p* = 0.04).

After adjusting for the effect of potential confounders, binary logistic regression analysis revealed that gender and geographical regions were the factors independently associated with physical and non-physical violence. For physical violence, female nurses had significantly lower odds of exposure compared to male nurses (OR = 0.42; 95% CI [0.21–0.83], *p* = 0.01), indicating that being female acts as a protective factor, decreasing the odds of experiencing physical violence. Regarding geographical distribution, nurses in the southern region demonstrated significantly lower odds of experiencing physical violence compared to those in other regions (OR = 0.62; 95% CI [0.40–0.97], *p* = 0.03), showing a reduction in the odds of exposure. Similarly, for non-physical violence, regional distribution remained significant, with the southern region showing a lower likelihood and decreased odds of exposure (OR = 0.56; 95% CI [0.37–0.87], *p* = 0.01), which represents a significant decrease in the odds of non-physical WPV (Tables 4 and 5).

The Hosmer-Lemeshow goodness-of-fit test was utilized to evaluate how well the models fit the data. While the physical violence model demonstrated a good fit (χ² = 3.25, *p* = 0.515), the Hosmer-Lemeshow test for the non-physical violence model yielded a p-value of 0.04, indicating a suboptimal fit. However, the overall model significance (*p* < 0.001) and the Nagelkerke R² of 34% support its explanatory utility despite this limitation. Furthermore, the Nagelkerke R² values indicate that the included predictors accounted for 47% of the variance in exposure to physical violence and 34% of the variance in non-physical violence, demonstrating a moderate to strong explanatory power.

**Table 4:** Factors significantly associated with physical violence

Variable	Physical violence					
	B	Wald	OR	95% C.I.		P value
				Lower	Upper	
Gender	-0.86	6.19	0.42	0.21	0.83	0.01*
Geographical regions	-0.47	4.31	0.62	0.40	0.97	0.03*
Model X^2	**38.99 (*p*** **< 0.001)**					
Nagelkerke R^2	**0.47**					
Hosmer-Lemeshow test.	**χ2(4) = 3.25**, ***p*** **= 0.515**					

P: significance level; χ2(df) = Chi-square statistic with degrees of freedom (df); B = unstandardized regression weight; OR = odds ratio; 95% C.I. = 95% confidence interval.*Statistically significant at *p* ≤ 0.05

**Table 5:** Factors significantly associated with non-physical violence

Variable	Non-Physical violence					
	B	Wald	OR	95% C.I.		P value
				Lower	Upper	
Geographical regions	-0.56	6.70	0.56	0.37	0.87	0.01*
Model X^2	**30.15 (** *p* **< 0.001)**					
Nagelkerke R^2	**0.34**					
Hosmer-Lemeshow test	χ2**(1) = 8.27**, ***p*** **= 0.04**					

P: significance level; χ2(df) = Chi-square statistic with degrees of freedom (df); B = unstandardized regression weight; OR = odds ratio; 95% C.I. = 95% confidence interval.*Statistically significant at *p* ≤ 0.05

#### Research Question 3

Are there differences in exposure to WPV against emergency nurses based on geographical location of hospitals (North, Middle, and South)?

There was a statistically significant difference between geographical regions and the exposure to physical violence (χ² = 6.112; *p* = 0.047), with the highest exposure observed among nurses in the Middle region (33.3%), followed by the North region (29.4%), while the South region reported the lowest exposure (15.3%). Similarly, a statistically significant difference was found regarding non-physical violence across the geographical regions (χ² = 15.755; p˂ 0.001). Similarly, a statistically significant difference was found regarding non-physical violence across the geographical regions(**χ²(2)** = 15.76, *p* < 0.001). Nurses in the Middle region experienced the highest proportion of non-physical violence (81.1%), followed by the North region (72.5%), whereas the South region reported the lowest rate (50.8%) (Table 6).

Nurses in the northern and middle regions were more vulnerable to WPV, with which a higher proportion of WPV was found in the middle region, then in the northern region (Table 6).

**Table 6:** The geographical regions differences in Jordanian hospitals towards WPV against emergency nurses

Geographical Region	North		Middle		South		χ2	*P*	df
Variables	*N*	(%)	*N*	(%)	*N*	(%)			
Exposed to physical violence							6.11	0.047*	2
Yes	15	29.4	30	33.3	9	15.3			
No	36	70.6	60	66.7	50	84.7			
Exposed to non-physical violence							15.75	0.001**	2
Yes	37	72.5	73	81.1	30	50.8			
No	14	27.5	17	18.9	29	49.2			
The impact of community culture on increased WPV							0.036	0.98	2
Yes	49	96.1	87	96.7	57	96.6			
No	2	3.9	3	3.3	2	3.4			
The methods used by the Jordanian community to cope with incidents after occurred							3.69	0.71	6
• The police have been notified to take action	24	47.1	33	36.7	20	33.9			
• Relatives of the aggressor give a solution	18	35.3	41	45.6	28	47.5			
• Inter-tribal settlement accord	6	11.8	10	11.1	9	15.3			
• Other	3	5.9	6	6.7	2	3.4			

P: significant level. χ2: chi-square. N: number %: percent df: degree of freedom

*Significant at the 0.05 level (2-tailed). **Significant at the 0.01 level (2-tailed)

## Discussion

### Workplace violence

According to surveys performed in other nations, including Malaysia Poland, Turkey, the Slovak Republic, and the Czech Republic and Spain, Jordan has the largest WPV against EDs [1, 12]. Although being the poorest in researches conducted in other nations like Iran, Egypt, Iraq, and Saudi Arabia [13–15]. The present study reveals that the incidence of WPV is 73.5%. Compared to other Jordanian studies, in 2017, the incidence of WPV in Jordan was 47.7% [6], while in 2021, it was 65.5% [16]. The WPV develops into a crisis that could prevent work from moving forward in all sectors, particularly the healthcare sector, which is primarily focused on saving the patient’s life. The WPV may delay this priority and increase the risk of mortality to the patients and staff [17].

The present study revealed that exposure to non-physical violence is higher than physical violence. This study agrees with other studies in Jordan, Malaysia, China, Brazil, Iran, Egypt, India, and Iraq. They reported that the incidence of non-physical violence is higher in more than 2–3 times than physical violence [1, 2, 14–16, 18, 19]. For this reason, having someone with leadership and communication skills to distress will be able to calm the environment.

The majority of violent incidents happened in the afternoon shift. When violent incidents occurred during the afternoon shift, similar survey results were obtained in many industrialised and developing countries [10, 20]. This may be due to that the afternoon is the time when most employees finish their work which increases the number of patients and patient partners, causing the ED to become crowded and healthcare providers to be unable to do their jobs properly, leaving patients and their families dissatisfied, which is enough to contribute to violence [21]. The recommendation is to increase the number of security staff in the ED during the afternoon and regulate the number of patient partners.

### The physical violence

In the past year, at least one episode of physical aggression was experienced by about a third of ED nurses. Research conducted in other nations revealed that physical violence there is less common than in Jordan. Brazil, Malaysia, Iraq, and India are among them [1, 14, 18, 19]. Even in Jordan, physical violence occurred around 16% of the time in the preceding year [16], although the prevalence of physical violence is higher than that of the current study in several other nations, including Egypt and Iran [2, 15]. High population numbers leading to more crowding in hospitals because the numbers may significantly reduce the absorptive capacity of hospitals, which increases the possibility of physical violence due to the inability to meet the patient’s needs [22].‏ To avoid this problem work should be done on building emergency clinics to decrease the crowd in ED in the hospitals.

### The non-physical violence

According to the results of the present research, more over two-thirds of individuals who were subjected to non-physical violence during the previous 12 months of emergency nurse practise had at least one such occurrence occur. When contrasted with countries like Egypt, Iraq, Brazil, and Malaysia, they were higher than Jordan [1, 2, 14, 18]. However, compared to the previous study in Jordan, the incidence of non-physical violence increased by approximately 28%, indicating that no serious action is being taken to address this challenge [16]. A total absence of interaction between patients and nurses may be the cause of the rise in the incidence of non-physical violence, unorganized nursing tasks, delay in giving medication, and unavailability of enough healthcare facilities, according to some research [23].

### The associated factors for WPV

Males were subjected to physical violence at a higher rate than females. This finding is congruent with the majority of the most recent studies involved in the WPV of these countries: Malaysia, Iran, and India (Saleh et al., 2020; Sushma et al., 2022; Zainal et al., 2018), even in Arab countries such as Egypt, Iraq, Saudi Arabia, and Jordan (Alsharari et al., 2021; Ghareeb et al., 2021; Hameed et al., 2020; Kabbash et al., 2019). This could be because, within society’s culture, men protect women. They do not harm them, and there is no physical contact between males and females in Arab society, even when they lose control of their anger, they can use non-physical violence to release it (Alshehri, 2017).

In this study, a higher number of emergency visitors, according to hospital capacity, were in the northern and middle regions, with which a higher proportion of WPV was found in the middle region, then in the northern region. More than two-thirds of participants reported being exposed to WPV. This finding is consistent with Abbas and Selim’s (2011) findings, which discovered that hospitals with a high number of patients had more incidents of WPV. The recommendation in this case to reduce exposure to violence in EDs is the increase in the number of hospitals and health facilities, the use of intelligent applications for booking appointments and an increase in the working time of outpatient clinics to cover unurgent cases instead of waiting in the emergency room and make it crowded.

### Factors associated with WPV

This survey suggests that prolonged treatment delays were the primary contributing factors to WPV, which is consistent with research from Jordan, India, and Palestine [16, 19, 24, 25]. Rather than acting as direct causes, long waiting times and hospital overcrowding are strongly associated with increased exposure to violence against emergency nurses. Owing to a staffing shortfall, a lack of emergency beds for the many patients who required therapy, and disorganized nursing tasks, lengthy wait periods create a high-stress environment that facilitates violent incidents. Such disruptions significantly hinder the continuity of the treatment process and compromise the delivery of safe, evidence-based nursing care [25].

The inability to address the requirements of the patients mates, which is related to a lack of nurses in the emergency department, is the second factor in physical violence in this study, according to Abbas and Selim. (2011) [26] reported that the inability to meet patient needs was caused by insufficient numbers of nurses, while Hameed et al. (2020) [24] reported that a shortage of nurses was the first factor associated with WPV.

The second factor contributing to on-physical violence was the unavailability of services or medications for patients which was linked to patient dissatisfaction. This finding agrees with Sadeh (2017) [27] study which reported that health quality programs applied and accreditation standards in the hospital must be applied according to the availability of medical services. The availability of products offered by hospitals that need to be developed and improved depends on monitoring patient satisfaction [28].

Psychiatric/Mental patients were identified in this investigation as the third factor of WPV. This is matched with Ghareeb et al. (2021) [16], Hameed et al. (2020) [14] and Sushma et al. (2022) [19].

### Geographical differences in Jordan toward WPV

The findings of the current study reveal that nurses in the northern and middle regions were more vulnerable to WPV. While, the Potential associated factor for WPV based on geographical area is that the northern region is more likely to develop WPV. This result was similar to Alshehri, (2017) [9] and Abbas and Selim’s (2011) [26] results, which found that a hospital with a high number of patients compared to its capacity was more exposed to WPV. These issues require immediate attention, such as medical visits to homes for early disease detection and treatment before complications and the need for emergency admission, this solution contributes to reducing the treatment cost, mortality rate, and hospital infrastructure [29].‏.

## Limitations of the study

Several limitations should be acknowledged in this study. First, the data were collected using self-reported questionnaires, which may introduce recall and reporting biases, as participants might over- or under-report experiences of WPV based on their subjective perceptions. Second, the study utilized a convenience sampling technique, which may limit the generalizability of the findings to all emergency nurses across different healthcare sectors in Jordan. Finally, the online data collection method might have introduced a potential response bias, as nurses with higher internet literacy or stronger feelings regarding workplace violence might have been more inclined to participate.

## Conclusion and recommendations

In conclusion, workplace violence is a prevalent issue among emergency nurses in Jordan, appearing to be closely associated with facility overcrowding and high patient volumes relative to hospital capacity. Notably, the Middle geographical region demonstrated a higher likelihood of exposure to both physical and non-physical violence compared to other regions. These violent incidents significantly disrupt the clinical workflow and the continuity of the treatment process.

To mitigate these risks, it is recommended that healthcare administrators consider structural strategies—such as extending outpatient clinic hours for non-urgent cases—to alleviate emergency department congestion, which is supported by the study’s findings on overcrowding. Furthermore, future research should explore the prevalence and contributing factors of WPV in private and military hospitals, and evaluate the effectiveness of specific preventive measures in minimizing the incidence and impact of workplace violence.

## Supplementary Information

Below is the link to the electronic supplementary material.

Supplementary Material 1

## Data Availability

The datasets used and analyzed during the current study are available from the corresponding author on reasonable request.

## Abbreviations

WPV: Workplace Violence
ED: Emergency Department
IRB: Institutional Review Board
MOH: Ministry of Health
SPSS: Statistical Package for the Social Sciences

## References

[1] Zainal N, Rasdi I, Saliluddin SM. The risk factors of workplace violence among healthcare workers in public hospital. Mal J Med Health Sci. 2018;14(SP2):120–7.

[2] Kabbash I, El-Sallamy R. Violence against health care workers in emergency hospital, Tanta University, Egypt. Egypt J Occup Med. 2019;43(2):215–28.

[3] Krug EG, et al. The world report on violence and health. lancet. 2002;360(9339):1083–8. 12384003 10.1016/S0140-6736(02)11133-0

[4] Lee H-L, et al. Workplace violence against emergency nurses in Taiwan: a cross-sectional study. J Emerg Nurs. 2020;46(1):66–71. 31733817 10.1016/j.jen.2019.09.004

[5] Darawad MW, et al. Violence against nurses in emergency departments in Jordan: Nurses’ perspective. Workplace health Saf. 2015;63(1):9–17. 25791406 10.1177/2165079914565348

[6] Higazee M, Rayan A. Consequences and control measures of workplace violence among nurses. J Nurs Health Stud. 2017;2(3):22.

[7] Galián-Muñoz I, et al. User violence and nursing staff burnout: The modulating role of job satisfaction. J interpers Violence. 2016;31(2):302–15. 25392390 10.1177/0886260514555367

[8] LoBiondo-Wood G, Haber J. Reliability and validity. G. LoBiondo, J. Haber, Nursing research: Methods and critical appraisal for evidence-based practice 2013: pp. 289–309.

[9] Alshehri FA. Workplace violence against nurses working in emergency departments in Saudi Arabia: a cross-sectional study. 2017;6(3):51–57.

[10] Kitaneh M, Hamdan M. Workplace violence against physicians and nurses in Palestinian public hospitals: A retrospective cross-sectional study. BMC Health Serv Res. 2012;12:1–8. 23256893 10.1186/1472-6963-12-469 PMC3541970

[11] Polit DF, Beck CT. Essentials of nursing research. Appraising evidence for nursing practice; 2014.

[12] Babiarczyk B, et al. Reporting of workplace violence towards nurses in 5 European countries-a cross-sectional study. Int J Occup Med Environ Health. 2020;33(3):325–39. 32235948 10.13075/ijomeh.1896.01475

[13] Alshehri FA. Workplace violence against nurses working in emergency departments in Saudi Arabia: A cross-sectional study. J Nurs Health Sci. 2017;6(3):51–7.

[14] Alhusain F, et al. Workplace violence against healthcare providers in emergency departments in Saudi Arabia. Saudi J Emerg Med. 2020;1(1):5–5.

[15] Saleh LA, et al. Relationship between workplace violence and work stress in the emergency department. J injury violence Res. 2020;12(2):183–93. 10.5249/jivr.v12i2.1526 32653889

[16] Ghareeb NS, El-Shafei DA, Eladl AM. Workplace violence among healthcare workers during COVID-19 pandemic in a Jordanian governmental hospital: The tip of the iceberg. Environ Sci Pollut Res. 2021;28(43):61441–9. 10.1007/s11356-021-15112-w PMC8233595 34173953

[17] Jia C, et al. Prevalence, characteristics, and consequences of verbal and physical violence against healthcare staff in Chinese hospitals during 2010–2020. J Occup Health. 2022;64(1):e12341. 35781909 10.1002/1348-9585.12341 PMC9262320

[18] Bernardes MLG, et al. Workplace violence among nursing professionals. Revista brasileira de Med do trabalho. 2020;18(3):250–7. 10.47626/1679-4435-2020-531 PMC7879475 33597974

[19] Sushma H, Nekar MS, Bant DD. Prevalence of Violence against Health Care Personnel in a Tertiary Care Centre, Hubballi, India. Natl J Community Med. 2022;13(02):79–84.

[20] Gacki-Smith J, et al. Violence against nurses working in US emergency departments. JONA: J Nurs Adm. 2009;39(7/8):340–9. 10.1097/NNA.0b013e3181ae97db 19641432

[21] Alkorashy HAE, Al FB, Moalad. Workplace violence against nursing staff in a Saudi university hospital. Int Nurs Rev. 2016;63(2):226–32. 26830364 10.1111/inr.12242

[22] Clery MJ, et al. Location of violent crime relative to trauma resources in Detroit: implications for community interventions. Western J Emerg Med. 2020;21(2):291–9. 10.5811/westjem.2019.9.44264 PMC7081851 31999248

[23] Jang SJ, Son YJ, Lee H. Prevalence, associated factors and adverse outcomes of workplace violence towards nurses in psychiatric settings: A systematic review. Int J Ment Health Nurs. 2022;31(3):450–68. 34773361 10.1111/inm.12951

[24] Hamdan M, Hamra A. Workplace violence towards workers in the emergency departments of Palestinian hospitals: a cross-sectional study. Hum Resour health. 2015;13(1):1–9. 25948058 10.1186/s12960-015-0018-2 PMC4435901

[25] Pich J, et al. Patient-related violence at triage: A qualitative descriptive study. Int Emerg Nurs. 2011;19(1):12–9. 21193163 10.1016/j.ienj.2009.11.007

[26] Abbas RA, Selim SF. Workplace violence: A survey of diagnostic radiographers in Ismailia governorate hospitals, Egypt. J Am Sci. 2011;7(6):1049–58.

[27] Sadeh E. Interrelationships among quality enablers, service quality, patients’ satisfaction and loyalty in hospitals. TQM J. 2017;29(1):101–17.

[28] Al-Mhasnah A, et al. The relationship between services quality and customer satisfaction among Jordanian healthcare sector. Manage Sci Lett. 2018;8(12):1413–20.

[29] Javanparast S, et al. Community health worker programs to improve healthcare access and equity: are they only relevant to low-and middle-income countries? Int J Health Policy Manage. 2018;7(10):943. 10.15171/ijhpm.2018.53 PMC6186464 30316247

