# Supplementary material for: The workplace violence against emergency nurses in Jordan: its impact on the treatment process and nursing work
Source: BMC Nurs. 2026 Aug 8;25:700. doi: 10.1186/s12912-026-05224-4 (PMC13459339; doi:10.1186/s12912-026-05224-4)
Supplement: Supplementary file 1 [file 12912_2026_5224_MOESM1_ESM.docx]

# Workplace Violence in the Health Sector Questionnaire

### Section A: Demographic Data

**Please answer the following questions by ticking the appropriate box or filling in the space:**

1. **Gender:** * [ ] Male
   - [ ] Female
2. **Age (years):** __________
3. **Education level (Qualification):**
   - [ ] Diploma
   - [ ] Bachelor’s degree
   - [ ] Master’s degree or higher
4. **Occupation:**
   - [ ] Registered Nurse
   - [ ] Head Nurse
   - [ ] Manager Nurse
5. **How long have you worked as a nurse (years)?** __________
6. **How long have you worked in the Emergency Department (years)?** __________
7. **Geographical Region of the Hospital:**
   - [ ] North
   - [ ] Middle
   - [ ] South

### Section B: Physical Workplace Violence

**Physical violence refers to the use of physical force against another person or group, which results in physical, sexual, or psychological harm.**

1. **Have you been physically attacked in your workplace in the past 12 months?**
   - [ ] Yes
   - [ ] No
2. **If yes, who was the attacker?**
   - [ ] Patient
   - [ ] Patient’s relative(s)
   - [ ] Staff member
   - [ ] Others
3. **At what time did the incident occur?**
   - [ ] Morning shift
   - [ ] Afternoon shift
   - [ ] Night shift

### Section C: Non-Physical Workplace Violence (Psychological)

**This includes verbal abuse, bullying/mobbing, and threats.**

1. **Have you been verbally abused (e.g., yelled at, insulted) in the past 12 months?**
   - [ ] Yes
   - [ ] No
2. **Have you been bullied or mobbed in the past 12 months?**
   - [ ] Yes
   - [ ] No
3. **Have you been verbally threatened in the past 12 months?**
   - [ ] Yes
   - [ ] No

### Section D: Protection Means and Community Culture

**This section identifies the systems of protection and the impact of society's culture on violence.**

1. **Are there specific policies or systems for protection against violence in your hospital?**
   - [ ] Yes
   - [ ] No
   - [ ] I do not know
2. **How did you respond to the incident of violence?**
   - [ ] Did nothing
   - [ ] Reported to supervisor/security
   - [ ] Told a colleague
   - [ ] Other: __________
3. **Does the community’s culture contribute to increasing workplace violence?**
   - [ ] Yes
   - [ ] No
   - [ ] I do not know
4. **In Jordanian society, what are the common procedures to cope with the incident after it has occurred?**
   - [ ] Relatives of the aggressor interfered and the personal right had waived.
   - [ ] The police had been notified to take action.
   - [ ] Inter-tribal settlement accord (Jaha/Sulh).
   - [ ] Others.
